# An Unusual Presentation of Invasive Group A *Streptococcus*—A Review of Epidemiology and Challenges in Management

**DOI:** 10.1155/crcc/6007143

**Published:** 2026-10-09

**Authors:** Fatima Hakak, Blake W. Jackson, Michael Addidle, Ulrike Buehner

**Affiliations:** ^1^ Department of Critical Care Rotorua Hospital Rotorua New Zealand nosm.ca; ^2^ Pharmacy Service Rotorua Hospital Rotorua New Zealand; ^3^ Pathlab NZ Tauranga New Zealand; ^4^ Department of Anaesthesia Rotorua Hospital Rotorua New Zealand

## Abstract

Invasive Group A *Streptococcus* (iGAS) infection is a disease state with increasing incidence internationally. This case report outlines a patient with severe septic shock from Group A beta‐haemolytic*Streptococcus* within a provincial intensive care unit (ICU) in New Zealand. Consequently, this case has prompted discussion on the local change in epidemiology for Group A beta‐haemolytic *Streptococcus* (GAS) and a review of the evidence of recognised interventions for iGAS and Streptococcal toxic shock syndrome (STSS).

## 1. Introduction

Group A beta‐haemolytic *Streptococcus* (GAS), also known as *Streptococcus pyogenes*, is a virulent gram‐positive pathogen responsible for a broad spectrum of disease states. GAS is responsible for not only simple cases of pharyngitis, but also life‐threatening and invasive conditions [1]. Invasive Group A *Streptococcus* (iGAS) is defined as the presence of GAS in a usually sterile site of the body such as blood, muscle or joint space [2, 3]. iGAS infections are often severe and can manifest in a variety of ways such as necrotising fasciitis or severe septicaemia. Streptococcal toxic shock syndrome (STSS) is a life‐threatening condition associated with multiorgan failure. STSS came to the forefront of medical attention in the late 1980s by groups such as Stevens et al. who described streptococcal infections with significant mortality [4]. The Centers for Disease Control and Prevention (CDC) diagnostic criteria for STSS involve hypotension; multiorgan failure characterised by two of renal impairment, coagulopathy, liver involvement, acute respiratory distress syndrome; and laboratory identification of GAS [5].

The epidemiology of iGAS has been of international interest, particularly following the COVID‐19 pandemic [6, 7]. New Zealand has historically had higher rates of iGAS infections compared with other high‐income countries. For example, in 2018 New Zealand had an iGAS rate of 9.4 cases per 100,000, whereas the United States was reported to have a rate of 7.6 per 100,000 [7]. The New Zealand Institute for Public Health and Forensic Science (PHF Science) in New Zealand has reported an increase in the incidence of iGAS infections in recent years. They demonstrated that iGAS infections rose to 10.9 cases per 100,000 in 2023, a steep rise compared with 4.8 cases per 100,000 in 2022 and 8.3 cases per 100,000 in 2019 (prior to the COVID‐19 pandemic) [8]. Although it is unclear which factors are driving the rise in iGAS infections, it has been speculated that low exposure to GAS during social isolation measures related to the COVID‐19 pandemic has reduced herd immunity to the organism itself. Since the publication of this data by PHF Science, iGAS has become a notifiable disease in New Zealand. With closer monitoring of such cases, it is hoped that the epidemiology of iGAS can be better understood to optimise the public health response [6].

The management of iGAS‐related sepsis is challenging, owing to the significant virulence of the organism itself. We would like to present a case of a patient that was managed in a provincial ICU in New Zealand who met the diagnostic criteria of iGAS, and subsequently STSS, and evaluate specific aspects of her inpatient management. The patient has provided both verbal and electronically registered consent for the presentation of this case.

## 2. Case Presentation

A 31‐year‐old New Zealand European woman presented to a provincial New Zealand Emergency Department feeling generally unwell the evening following a routine haemorrhoidectomy. She was found to be febrile (39.3°C), centrally cyanotic, tachycardic (120–130 beats per minute) and profoundly hypotensive (mean arterial pressure (MAP) 43 mmHg) requiring vasopressor support. The patient was of a normal body mass index, had one previous normal vaginal delivery and was fit and well otherwise.

On admission, she received aggressive fluid resuscitation, noradrenaline support, a stat dose of intravenous (IV) gentamicin and was started on IV cefuroxime and metronidazole once an abdominal source of infection was suspected.

Despite the above resuscitative measures, the patient required dramatic escalation of therapy during the first 12 h of admission. The patient became increasingly hypotensive (MAP 50) and tachycardic (140–150 beats per minute). In turn, she had an escalating requirement of noradrenaline necessitating the addition of vasopressin and hydrocortisone. Simultaneously, a blood culture flagged positive after 15 h incubation. Gram stain revealed the presence of gram‐positive cocci. Through consultation with our ICU pharmacist and clinical microbiology team, it was agreed to broaden her antibiotic therapy to IV piperacillin‐tazobactam. Because of concerns that the haemorrhoidectomy wound may be fragile and susceptible to infection, the patient had acute surgery to create a diversion colostomy. The patient returned to the ICU postsurgery intubated with a high‐set respiratory rate to correct the significant metabolic acidosis in keeping with the burden of septic shock. The severity of metabolic acidosis during the first day of admission was reflected in a pH of 7.1, base excess of −13.5 mmol/L and lactate of 4.9 mmol/L.

With concern that the pathogen may possibly be a beta‐haemolytic *Streptococcus*, the patient was started on IV clindamycin on return from surgery, with the view of downregulating possible toxin production [9]. Organism identification was achieved by the next morning, almost 30 h into admission, which confirmed a GAS bacteraemia susceptible to penicillin and clindamycin. This organism was also later identified from a peritoneal fluid sample collected in theatre. By this stage, the patient was meeting criteria for STSS as she was hypotensive, demonstrated renal impairment and acute respiratory distress syndrome, and had GAS identified on a laboratory sample. IV piperacillin‐tazobactam and IV clindamycin were continued. As a result of learnings from the BLING‐III Trial [10], it was elected for her antibiotics to be run as a continuous infusion to optimise her sepsis management. Discussion regarding the potential role of intravenous immunoglobulin (IVIg) took place with the clinical microbiology team. However, as she was improving both clinically and biochemically following her surgery, it was elected not to pursue IVIg as part of her management.

This patient spent a total of 7 days in the ICU. She was extubated on the evening of “Day 2” but remained in the unit for blood pressure optimisation, fluid management and management of sepsis‐related delirium. This patient has recovered well and has already achieved the reversal of her colostomy with a good outcome.

## 3. Discussion

A notable feature of this case was the identification of GAS in both blood cultures and peritoneal fluid. This pathogen came as a surprise, as it was not in keeping with the pathogens one would expect following lower gastrointestinal surgery. The group postulates that GAS was introduced to the haemorrhoidectomy wound, subsequently causing an invasive and ascending infection through the abdomen. When considering management of this patient, key management issues that arose were the use of continuous antibiotic administration in sepsis and the role of IVIg in severe iGAS and STSS.

Continuous versus intermittent antibiotic administration has been a contentious topic amongst critical care colleagues. It is accepted that the bactericidal quality of beta‐lactam antibiotics is time‐dependent and thus their clinical effectiveness is thought to be influenced by the duration of the drug concentration above the minimum inhibitory concentration (MIC) of the target organism [11]. At the time of this patient encounter, the *Surviving Sepsis Campaign* guidelines published in 2021 also supported the sentiment that prolonged infusions of beta‐lactams should be used for septic adults. This suggestion was regarded as “a weak recommendation with a moderate quality of evidence” [12]. Importantly, the most recent edition of the *Surviving Sepsis Campaign* published in 2026 has revisited this management principle. The guideline now advises for adults with sepsis‐prolonged infusion of beta‐lactams for maintenance is a “strong recommendation” with “moderate certainty evidence” [13]. Multiple publications between the release of the 2021 and 2026 guidelines have contributed to this firmer recommendation. In particular, Dulhunty et al. [10] conducted the BLING‐III randomised clinical trial (RCT) comparing continuous and intermittent dosing of IV piperacillin‐tazobactam and meropenem in sepsis. They found a greater clinical cure using continuous antibiotic infusions compared with intermittent infusions (55.7% vs. 50.0%, respectively). Abdul‐Aziz et al. also completed a systematic review of RCTs on this topic [14]. This research group found that the use of prolonged beta‐lactam antibiotic infusions was associated with a reduced risk of 90‐day mortality compared with intermittent infusions. It was also determined that continuous infusions were associated with a reduced risk of ICU mortality (“high certainty”) and an increase in clinical cure (“moderate certainty”). Li and Jiang evaluated Abdul‐Aziz et al.′s meta‐analysis and the BLING‐III trial and submitted their commentary to *Critical Care* in November 2024. They expressed an overall positive acceptance of prolonged infusions of beta‐lactam antibiotics, demonstrating that the results of the BLING‐III trial in fact supplement rather than conflict with meta‐analyses thus far [11].

IVIg therapy in the context of iGAS sepsis has been explored by several research groups. IgG molecules exhibit bactericidal effects mediated by several mechanisms including complement, toxin inactivation and opsonisation and viral neutralisation [15]. As such, it is believed that IVIg can also initiate these immunomodulatory mechanisms in severe disease states. A Cochrane review conducted by Alejandria et al. reviewed RCTs using IVIg in patients with bacterial sepsis or septic shock. They found polyclonal IVIg reduced mortality in septic adult patients; however, this was not seen in trials with low risk of bias [16]. The *Surviving Sepsis* 2021 guideline suggests against using IVIg (weak recommendation, low quality of evidence). On evaluation of existing evidence, the balance of beneficial and unwanted effects of IVIg remain unclear and given the significant expense of IVIg, it may not provide appropriate benefit to such patients [12]. There has been no change to this recommendation in the 2026 edition of *Surviving Sepsis* [13]. The New Zealand Blood Bank refers clinicians to the Ig governance clinical tool for assessing the indications of IVIg. They state that although evidence for IVIg in STSS is variable, given the severity of disease that can result from this syndrome and some positive benefits identified for IVIg use in these cases of sepsis, IVIg can be considered safe to use as an adjunctive therapy [17]. Ultimately, further clarity is required to understand what role IVIg can contribute to the management of STSS.

## 4. Conclusion

There is now an irrefutable trend demonstrating an increase in iGAS infections globally. Having presented with sepsis following a gastrointestinal surgery, GAS was not immediately suspected to be the causative organism in this patient case. The unusual presence of iGAS in the context of an abdominal pathology demonstrates the pervasiveness of iGAS currently in New Zealand and serves as an example to the critical care community to consider GAS as a pathogen in all severely septic patients. The virulence of GAS poses a significant challenge in maintaining haemodynamic stability in critical care patients and thus warrants serious consideration of continuous IV beta‐lactam therapy with the current evidence available. The role of IVIg remains controversial, thus warranting further research to determine if its use provides benefit to patients with severe iGAS.

## Funding

No funding was received for this manuscript.

## Conflicts of Interest

The authors declare no conflicts of interest.

## Data Availability

Data sharing is not applicable to this article as no datasets were generated or analysed during the current study.
